# Low blood flow velocity in the left atrial appendage in sinus rhythm as a predictor of atrial fibrillation: results of a prospective cohort study with 3 years of follow-up

**DOI:** 10.1186/s42466-025-00381-4

**Published:** 2025-04-14

**Authors:** Gero Klinger, Lea Schettler, Greta Schettler, Mathias Bähr, Gerd Hasenfuß, Mark Weber-Krüger, Jan Liman, Marlena Schnieder, Marco Robin Schroeter

**Affiliations:** 1 Department of Neurology, University-Medical-Center Göttingen, Göttingen, Germany; 2 Heart Center, Department of Cardiology and Pneumology, University Medical Center Göttingen, Göttingen, Germany; 3 Department of Neurology, Paracelsus Medical Private University Klinikum Nuremberg, Nuremberg, Germany

**Keywords:** Atrial fibrillation, Left atrial appendage, Transesophageal echocardiography, Ischemic stroke

## Abstract

**Background:**

Atrial fibrillation (AF) is a common cause of cardioembolic stroke and can lead to severe and recurrent cerebrovascular events. Thus, identifying patients suffering from cardioembolic events caused by undetected AF is crucial. Previously, we found an association between increasing stroke severity and a decreasing left atrial appendage (LAA) blood flow velocity below 60 cm/s.

**Methods:**

This was a prospective single-center cohort study including hospitalized patients who underwent a transesophageal echocardiography (TEE) in sinus rhythm. The participants were divided into two groups (*≥* 60 cm/s;<60 cm/s) based on their maximum LAA blood flow velocity. The results of the cardiovascular risk assessment and 24- to 72-hour ECG Holter were recorded. Follow-up appointments were scheduled at 3, 6, 12, 24 and 36 months. The primary endpoint was new-onset AF. The statistics included a Cox-proportional-hazard-model and a binary logistic regression. Numerical data or categorical data were analyzed with the Mann-Whitney U test or chi-square test.

**Results:**

A total of 166 patients were recruited. The median LAA blood flow velocity was 64 cm/s. New-onset AF was diagnosed in 22.9% of the patients. An LAA blood flow velocity *≤* 60 cm/s was associated with a threefold increased risk of new-onset AF (35.8% vs. 11.5%; HR3.56; CI95%1.70–7.46; *p* < 0.001), independently according to a multivariate analysis (*p* = 0.035). Furthermore, a decreasing LAA blood flow velocity was associated with an increased risk of new-onset AF (OR1.043; CI95%1.021–1.069; *p* < 0.001).

**Conclusion:**

A **l**ow LAA blood flow velocity (*≤* 60 cm/s) in sinus rhythm is prospectively associated with an increased risk of new-onset AF. Additional simple LAA-TEE examinations could help to identify patients who benefit from more accurate cardiac rhythm monitoring.

## Introduction

Atrial fibrillation (AF) is the most common arrhythmia with increasing incidence and prevalence in the elderly [1–3]. The incidence of ischemic stroke is five times greater in patients with AF [4] and it is therefore a common cause of cardioembolism [5] due to ineffective left atrial contraction and blood stasis, leading to thrombus formation [6]. If a cardiac thrombus is identified, it typically appears in the left atrial appendage (LAA) [7]. In patients with cryptogenic stroke, prolonged cardiac rhythm monitoring or loop recorders demonstrated a higher detection rate of AF compared to standard diagnostics methods. However, they did not investigate if a higher AF detection rate can improve secondary stroke prevention [8–11]. Whether prolonged rhythm monitoring in stroke patients leads to a better secondary stroke prevention is a current topic of research [12]. For this reason, there is a need for biomarkers to identify patients at high risk of new-onset AF to apply intensive rhythm monitoring approaches that are more tailored.

Previously, we retrospectively demonstrated that a decreasing LAA blood flow velocity below 60 cm/s was associated with an increasing stroke severity regardless of the heart rhythm [13]. Others have demonstrated a negative association between LAA blood flow velocity and multiple cerebral infarcts [14]. While a low LAA-blood-flow velocity usually occurs in patients with AF [15, 16], the role of low LAA blood flow velocity in sinus rhythm may require further attention. Therefore, we wanted to determine the role of a low LAA blood flow velocity (< 60 cm/s) in sinus rhythm as a predictor of occurring or hidden atrial fibrillation.

## Methods

### Screening, design and follow-up

This prospective observational cohort study included patients over 60 years of age, excluding patients with AF, atrial flutter, an oral anticoagulation or an LAA occlusion device. Transesophageal echocardiography (TEE) performed in sinus rhythm was used as the primary screening parameter. TEE was used to measure the maximum accessible blood flow velocity at the left atrial appendage’s orifice via pulsed-wave Doppler sonography over at least 3–4 atrial contraction cycles. Other parameters reported were cardiac valve status, intracardiac thrombus or spontaneous echocardiographic contrast or a patent foramen ovale. Furthermore, a medical history was evaluated, including a history of stroke, cardiovascular disease and internal organ dysfunction. The amino-terminal pro-brain-natriuretic-peptide (NT-proBNP) serum concentration was obtained from the laboratory database. The CHA2DS2-VASc score was calculated for each patient. The participants underwent a transthoracic echocardiography (TTE) and had a 24- to 72-hour ECG Holter. Transesophageal and transthoracic echocardiography procedures were performed via IE33, CX50, and X7-2t probes (Philips Medical Systems, Eindhoven, Netherlands) or Vivid E9 and 6VT-D probes (GE Healthcare, USA). Follow-up appointments were arranged by telephone after 3, 6, 12, 24 and 36 months with an assessment to determine whether a primary endpoint– new-onset AF– or a secondary endpoint– new stroke after AF diagnosis or death– had been accomplished. Detected AF with an ECG-holter at baseline was considered as a primary endpoint reached within 3 months. In patients with a cardiac intervention, new-onset AF was then stated as potential postinterventional AF, if it was found in the long-term ECG-holter.

### Statistics

This prospective design is based on our previous retrospective study that included patients with a transient ischemic attack or an ischemic stroke who underwent a TEE examination between 2012 and 2014 (3 years). We primarily found that a decreasing blood flow velocity in the LAA was associated with an increasing stroke severity at a threshold below 60 cm/s for the LAA blood flow velocity. A total of 25.9% of the patients with a low LAA blood flow velocity < 60 cm/s (LAA-LF) had prevalent AF compared to 7.5% with a high LAA blood flow velocity *≥* 60 cm/s (LAA-HF) [13]. We therefore expected 25.9% cases of new-onset AF in our prospective study group (LAA-LF; < 60 cm/s) vs. 7.5% in our control group (LAA-HF; *≥ *60 cm/s) within 3 years. We calculated that 127 participants would be required to achieve 80% statistical power. At this point, we expected that an explorative data analysis could first yield results of statistical and clinical relevance.

Descriptive characteristics are presented via the Mann-Whitney U test. The chi-square test identified differences between categorical variables. We examined LAA-LF for its possibility of being an independent predictor for new-onset AF via a multivariate analysis. A Cox-proportional-hazard-model was used to calculate differences in the frequencies via the log-rank test. A binary logistic regression was performed to examine the continuous dependency between the LAA blood flow velocity at inclusion to this study and new-onset AF during the observation time in patients without stated postinterventional AF. Based on the binary logistic regression, we created a receiver operating characteristics (ROC) curve to identify the LAA blood flow velocity threshold with the best test accuracy. Positive and negative predictive values were calculated by using the overall AF prevalence in our prospective sample. With Fisher´s exact test, we evaluated if patients with new-onset AF suffered more often from stroke or death. Statistics were performed with IBM SPSS Statistics (version 28.0.1.) and GraphPad Prism 10 (version 10.1.1.). The level of significance was defined as *p* = 0.05.

## Results

### Baseline characteristics

**Fig. 1:**
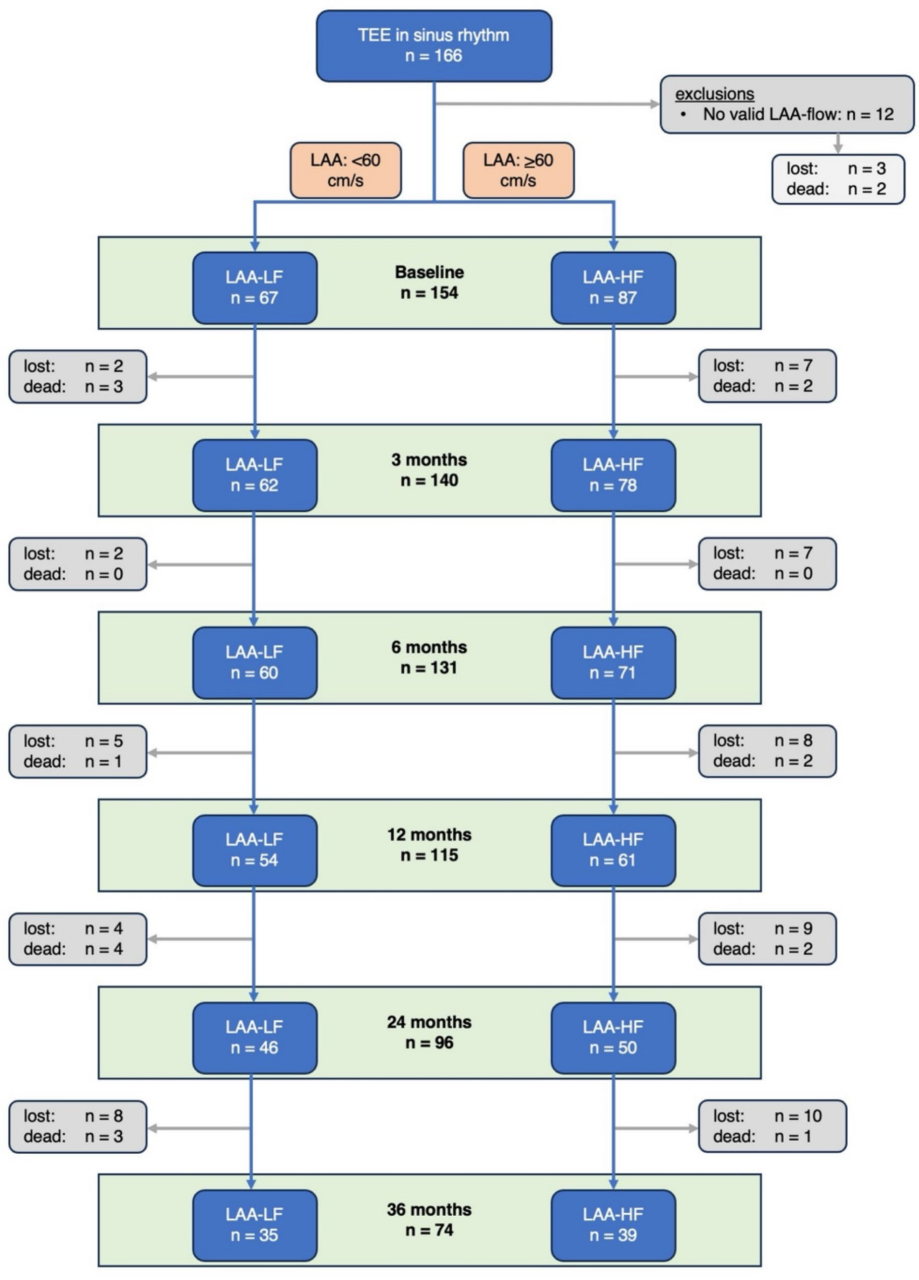
Flowchart of the recruitment and follow-up process. Abbreviations: AF, atrial fibrillation, LAA-LF, left atrial appendage low blood flow velocity < 60 cm/s; LAA-HF, left atrial appendage high blood flow velocity *≥* 60 cm/s, TEE, transesophageal echocardiography

Between November 2018 and November 2023, a total of 166 participants were included in our study (Fig. 1). The median LAA blood flow velocity was 64 cm / s (IQR 33). The median age was 73 (IQR 16) years, and 44.6% were female.

When LAA-LF patients were compared with LAA-HF patients (Table 1), those with LAA-LF were older (76 vs. 68 years; *p* < 0.001), were more likely to suffer from coronary artery disease (56.7% vs. 33.3%; *p* = 0.009) and tended to have a chronic kidney disease (CKD) more frequently (19.4% vs. 8.0%; *p* = 0.052). A higher CHA2DS2-VASc score was more common in LAA-LF patients (5 vs. 4 points; *p* = 0.035). Most of the participants were hospitalized for transfemoral aortic valve replacement (TAVR; 38.0%) or with acute ischemic stroke (43.4%) at enrollment. Less frequent were a surgical aortic valve replacement (4.2%), a percutaneous coronary intervention (2.4%), a transcatheter edge-to-edge repair of the mitral valve (2.4%) or other reasons (9.6%). From the patients hospitalized with acute ischemic stroke at inclusion to this study, 36% were either caused by an ESUS or have been classified cryptogenic, 33% were not further classified, 11% had a cardioembolic etiology and 20% were based on a microangiopathic, macroangiopathic or a special cause.

**Table 1:** Baseline characteristics and cardiovascular diseases. Categorical data are presented with absolute (n) and relative (%) values to all participants, LAA-LF and LAA-HF participants. Numerical data are presented as medians and interquartile ranges (IQRs)

Descriptive data	All *n* = 154	LAA-LF < 60 cm/s *n* = 67	LAA-HF *≥* 60 cm/s *n* = 87	*p*
Median Age (years)	73 (IQR16)	76 (IQR13)	68 (IQR16)	< 0.001
Male	86 (55.8%)	35 (52.2%)	51 (58.6%)	0.41
Median Body Mass Index (points)	27 (IQR4)	26 (IQR5)	27 (IQR5)	0.31
Hypertension	123 (79.8%)	58 (86.6%)	65 (74.7%)	0.12
Dyslipidemia	96 (62.3%)	38 (56.7%)	58 (66.7%)	0.24
Smoking	66 (42.8%)	31 (47.0%)	35 (41.2%)	0.51
Diabetes mellitus type	43 (27.9%)	21 (31.3%)	22 (25.6%)	0.47
Coronary artery disease	67 (43.5%)	38 (56.7%)	29 (33.3%)	0.009
Congestive heart failure	24 (15.6%)	14 (20.9%)	10 (11.5%)	0.18
Chronic kidney disease	20 (12.9%)	13 (19.4%)	7 (8.0%)	0.052
Myocardial infarction	20 (12.9%)	11 (16.4%)	9 (10.3%)	0.23
Peripheral arterial disease	11 (7.1%)	5 (7.5%)	6 (6.9%)	1.0
Previous ischemic stroke	75 (48.7%)	24 (35.8%)	51 (58.6%)	0.002
Median CHA2DS2-VASc-Score (points)	4 (IQR2)	5 (IQR2)	4 (IQR2)	0.035

### Primary and secondary outcomes

**Fig. 2:**
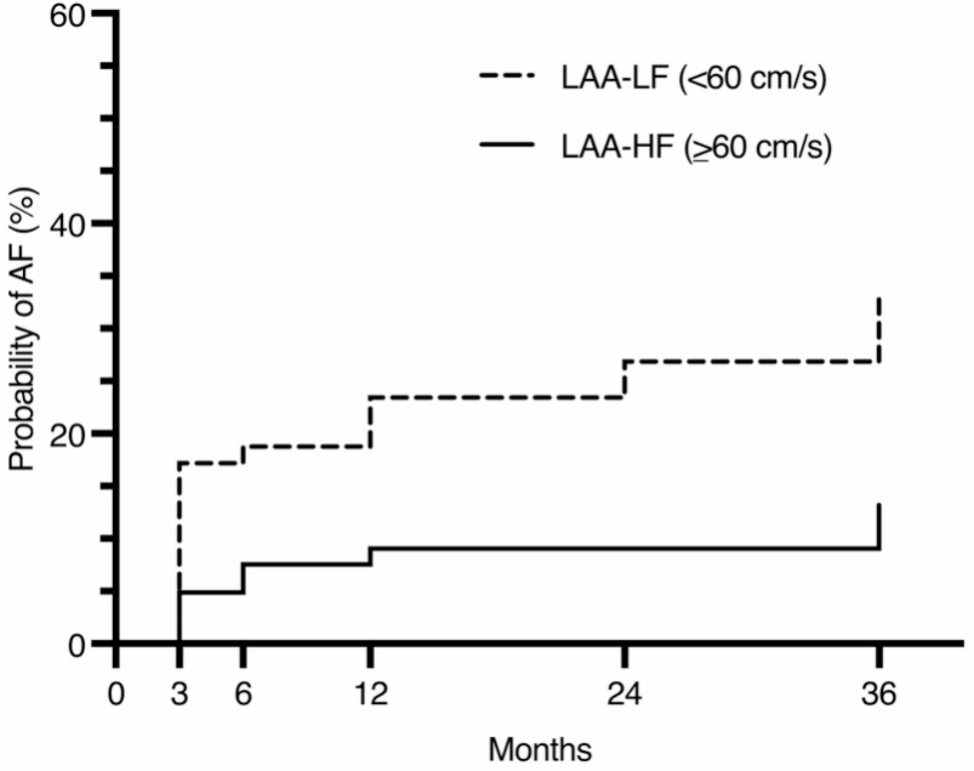
Cox-proportional hazard model of LAA-LF and LAA-HF patients and new-onset AF probability in over 3 years of observation

A total of 38/166 (22.9%) of the participants in this study were diagnosed with AF during the observation period. Four patients had no valid LAA blood flow velocity measurements and three patients were stated with postinterventional AF and have been excluded from further analysis. In a multivariate analysis (Table 2), we found that LAA-LF was an independent risk factor for new-onset AF (OR1.052; CI95%1.003–1.103; *p* = 0.036). Moreover, we indicated that patients with LAA-LF in sinus rhythm have a threefold increased risk of being diagnosed with new-onset AF (35.8% vs. 11.5%; HR3.56; CI95%1.70–7.46; *p* < 0.001) (Fig. 2).

**Table 2:** Comparison of risk factors associated with new-onset AF in a multivariate analysis. Abbreviations: LAA, left atrial appendage; LVEF, left ventricular ejection fraction; LAVI, left atrial volume index; BMI, body mass index; NT-proBNP, aminoterminal pro-brain-natriuretic peptide

Risk factor	Adjusted odds ratio	95% confidence interval		*p*
LAA blood flow velocity < 60 cm/s (*n* = 154)	1.052	1.003	1.103	0.036
LVEF (*n* = 146)	1.002	0.921	1.089	0.966
LAVI (*n* = 104)	1.025	0.979	1.073	0.3
Age (*n* = 166)	1.060	0.950	1.182	0.29
BMI (*n* = 166)	1.068	0.887	1.285	0.49
Sex (male; *n* = 92)	0.408	0.083	1.083	0.27
Coronary artery disease (*n* = 70)	2.253	0.342	14.854	0.4
Chronic kidney disease (*n* = 22)	0.73	0.105	5.078	0.75
Congestive heart failure (*n* = 24)	1.607	0.260	9.932	0.61
NT-proBNP (*n* = 73)	1.0	1.0	1.0	0.4

**Fig. 3:**
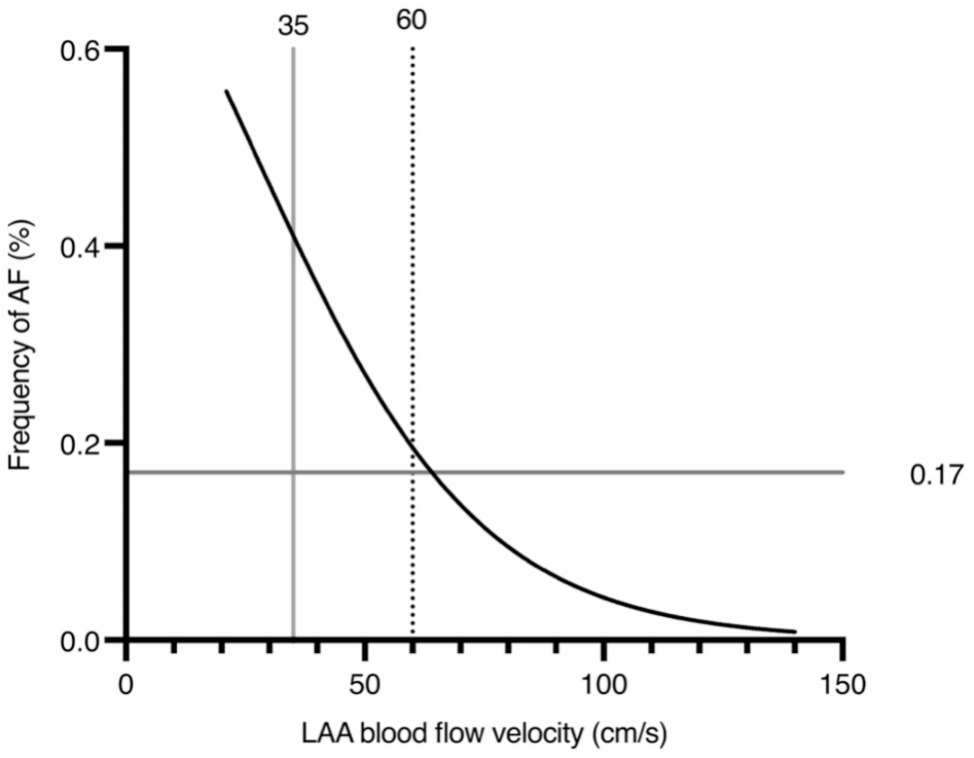
Binary logistic regression plot of LAA blood flow velocity at inclusion to this study and the new-onset AF frequency during the observation time

Figure 3 shows that a decreasing LAA blood flow velocity below 60 cm/s by 1 cm/s is associated with an increased risk of 4.3% for new-onset AF (OR1.043; CI95%1.021–1.069; *p* < 0.001). At an LAA blood flow velocity of < 35 cm/s, the chance for new-onset AF increases to > 40%.

**Fig. 4:**
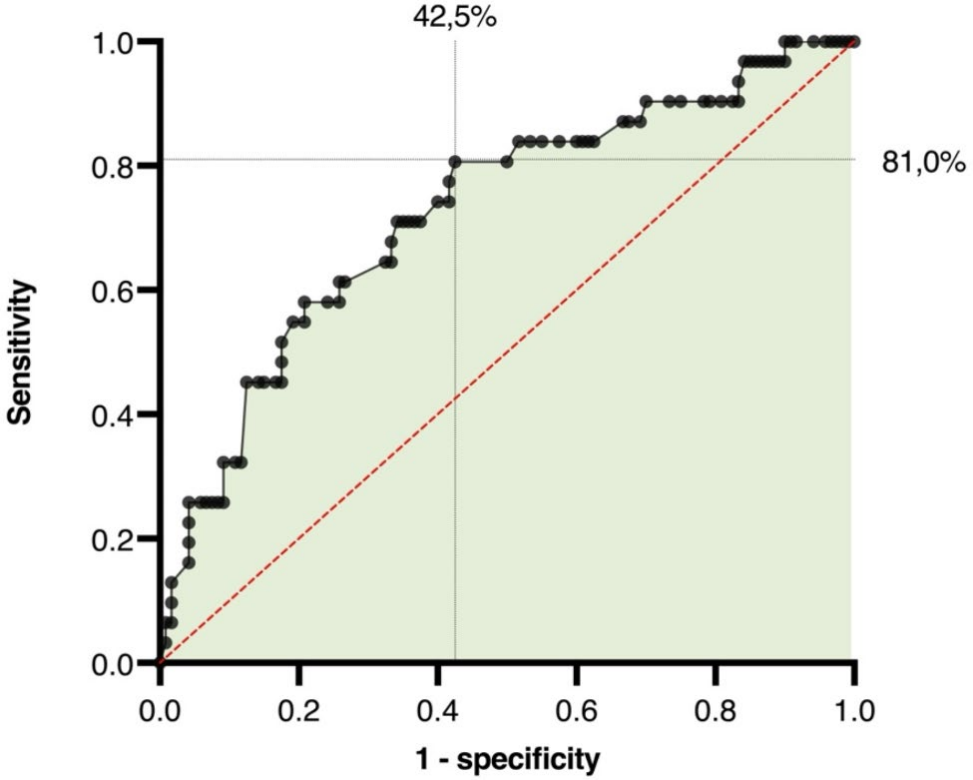
Receiver operating characterstics curve of the LAA blood flow velocity as a predictor of new-onset AF

According to the ROC curve and Youden’s index, the optimal threshold of the LAA blood flow velocity, which can separate patients at risk for new-onset AF from those without increased risk, was found to have a sensitivity and specificity of 81% and 58% (Fig. 4) and a chance for new-onset AF of *≥* 17% in the binary logistic regression model (AUC-ROC 0.726; CI95%0.624–0.829; *p* < 0.001). At this cut-off value, we found that an LAA blood flow velocity threshold of 65 cm/s can distinguish patients at high risk for new-onset AF from those without it. The negative predictive value (NPV) at this threshold was 91%, whereas the positive predictive value (PPV) was 36% based on the overall AF prevalence in our data.

With respect to our secondary endpoints, a new ischemic stroke occurred eight times in patients with new-onset AF (one after 3 and 6 months, two after 12 months, and four after 36 months) and eight times in patients without new-onset AF (three after 3 months, two after 6 and 12 months, and one after 24 months). Therefore, patients with new-onset AF were more likely to suffer from a new stroke prospectively (21.1% vs. 6.1%; OR4.067; CI95%1.348–12.176; *p* < 0.05). Early strokes after 3 months occurred only in patients initially included in this study with acute ischemic stroke. Therefore, early strokes were not suggested as postprocedural. Eighteen patients died during the observation time, whereas participants with an LAA-LF died twice as often without statistical significance (16.4 vs. 8.0%; OR2.245; CI95%0.800–5.768; *p* = 0.109).

### Echocardiographic, electrocardiographic and laboratory findings

**Fig. 5:**
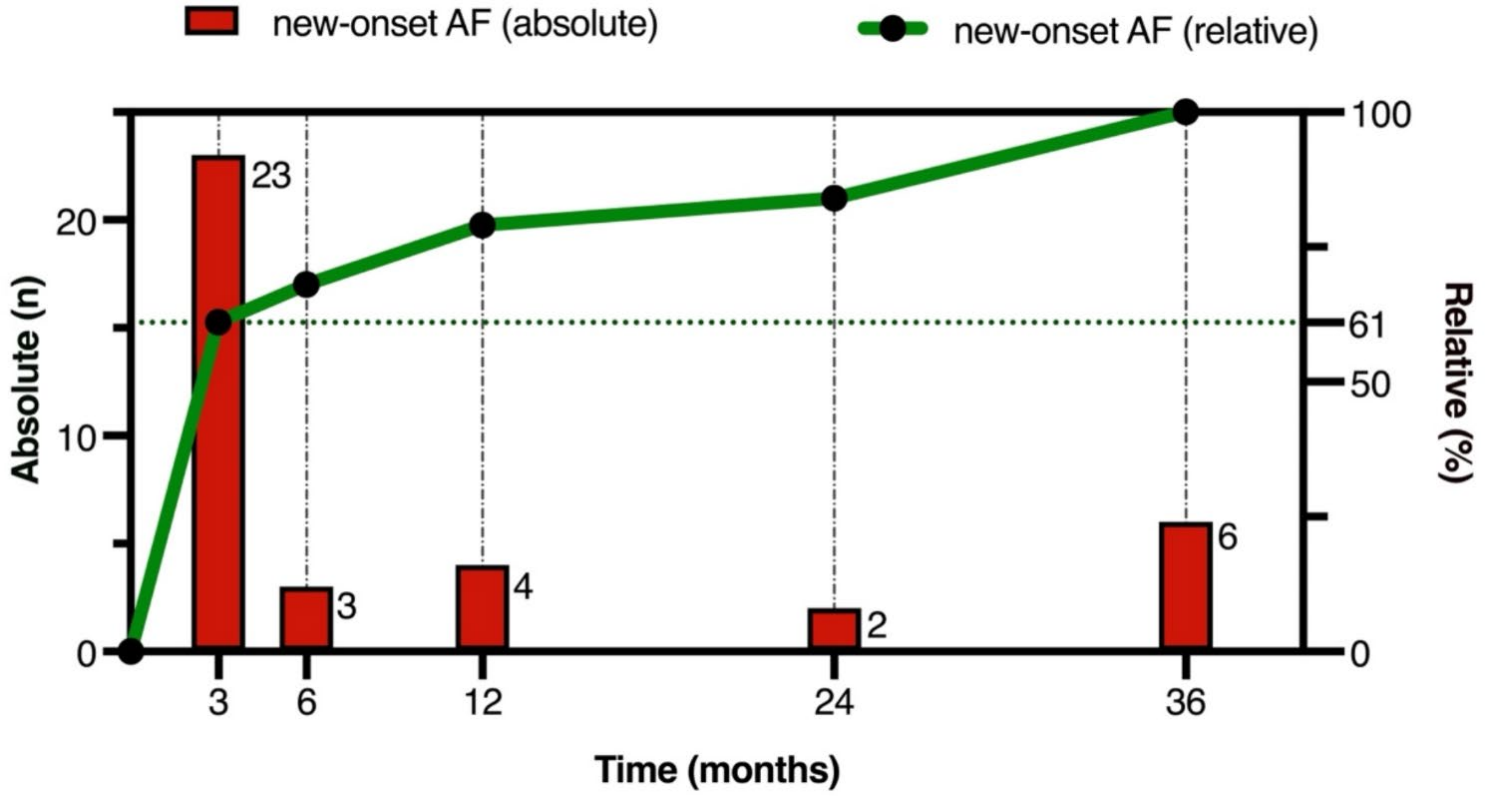
Number of patients with new-onset AF in absolute (red) and relative (green) values according to the several follow-up appointments. AF detection in the long-term ECG-holter was included in the first three months of observation

Most patients had a normal left ventricular ejection fraction (LVEF) of 55% (IQR0). NT-proBNP in 73 participants was significantly different between LAA-LF and LAA-HF patients (1082 vs. 515 ng/L; *p* = 0.018). The left atrium volume index in 104 was greater in patients with LAA-LF (38 vs. 47 mL/m^2^; *p* < 0.001). Data from 24- to 72-hour ECG-Holter recordings were available from 139 (83.7%) patients with a median detection time of 46 h (IQR 37), among whom 7 patients experienced AF after undergoing TEE in sinus rhythm. After 12 months of observation, 30 out of 38 (79%) of all patients with new-onset AF had already received their diagnosis (Fig. 5). Seven patients (18.4%) were initially diagnosed by the long-term ECG-Holter with three patients after a surgical (one) or transfemoral (two) aortic valve replacement, three patients after an ischemic stroke and one patient after a urogenital infection. In our study, severe aortic valve stenosis with TAVR was not associated with new-onset AF (*p* = 0.09) or LAA-LF (*p* = 0.065).

## Discussion

### Primary outcome

In this prospective single-center observational study, we observed that patients with an LAA blood flow velocity < 60 cm/s in sinus rhythm had an independently increased risk of new-onset AF within 3 years of observation. The likelihood of new-onset AF increased with decreasing LAA blood flow velocity. In addition, new-onset AF was associated with a higher rate of new strokes.

According to the ROC curve, the highest test accuracy was found at a threshold of < 65 cm/s LAA blood flow velocity with a sensitivity of 81% and specificity of 58%. This indicates a small PPV for LAA-LF patients, but therefore a high NPV for LAA-HF patients and a LAA blood flow velocity of > 65 cm/s of 91%. The specificity exceeds 94% for an LAA blood flow velocity < 35 cm/s. Consequently, another study with stroke patients reported an NPV of 100% for LAA thrombi at an LAA blood flow velocity of *≥* 55 cm/s. An LAA blood flow velocity of < 55 cm/s was associated with LAA thrombi regardless of the heart rhythm and the likelihood of an LAA thrombus was increasing with a decreasing blood flow velocity [17]. While an LAA blood flow velocity < 60 cm/s was also associated with an increasing stroke severity regardless of the heart rhythm [13], our findings could support a thesis of a higher incidence of masked AF at low LAA blood flow velocities, resulting in LAA thrombosis and a greater risk of cardioembolic stroke. Moreover, LAA morphology is heterogeneous, and different morphologic subtypes have already been associated with stroke elsewhere [18]. Therefore, additional TEE examination could provide not only functional but also morphological information about the LAA in patients with recent stroke. Additional LAA-TEE data could be integrated into a rule-in (LAA-LF) or rule-out (LAA-HF) strategy, to decide between prolonged / invasive cardiac rhythm monitoring (LAA < 60 cm/s) or standard care (LAA *≥* 60 cm/s) to search for undetected AF. Nonetheless, our calculations of the test accuracy for different cut-off values of the LAA blood flow velocity for the prediction of new-onset AF must be tested for validity and reliability in further studies with more specified and homogeneous participants.

We found that approximately 80% of all participants with new-onset AF were diagnosed during the first 12 months of observation. The diagnosis was either based on the results of a single long-term ECG-holter measurement at admission to this study or the telephone interview at each follow-up appointment. From the seven patients with new AF in the ECG-holter, three had a cardiac intervention at inclusion to this study. These patients could have suffered from postinterventional AF which is known to be 10% in patients after TAVR and 33% after SAVR [19]. Therefore, the influence of the LAA blood flow velocity remains unclear in these participants. Moreover, less than half of the participants have reached the 36-month follow-up appointment. The new-onset AF incidence and detection likelihood could therefore be overestimated within the first three months or underestimated after 3 years of observation. Compared with the average annual incidence for AF, which is known to be 1% and increases to 2% per year in patients aged 80 years [20], we observed higher rates of new-onset AF in both our groups, leading to an overall incidence of 22.9% over 3 years (19% in one year). In the ATTICUS trial, most of the patients with new AF were diagnosed 4 months after the index stroke [21], and in the CRYSTAL-AF trial, 18% of the participants with insertable cardiac monitors had new AF found about one year after enrollment [8]. These findings could be explained by high selected samples of hospitalized patients who are at greater risk of AF compared to the general population.

### Cardiovascular risk, atrial fibrillation, stroke and left atrial cardiopathy

LAA-LF patients were older and had a greater prevalence of coronary artery disease (CAD), elevated NT-proBNP levels, left atrial enlargement and cardiovascular burden, as reflected by higher CHA2DS-VASc-scores and a trend toward chronic kidney disease (CKD). Left atrial enlargement and elevated NT-proBNP serum concentrations have already been associated with new-onset AF in patients with ischemic stroke [22–24]. In our multivariate model, they did not present an influence on new-onset AF which might be explained be a high number of patients with a measured LAA blood flow velocity compared to a low number of patients with available data on LAVI or NT-proBNP results. Older age was already associated with a higher incidence of AF elsewhere [25] and older patients demonstrated a benefit in receiving dabigatran over aspirin after an ESUS [26]. Moreover, coronary endothelial dysfunction was associated with an increased risk of AF in another study [27]. Others have demonstrated a negative correlation between the CHA2DS2-VASc score and LAA blood flow velocity [16], whereas a CHA2DS2-VASc score of at least 5 points has already been associated with stroke regardless of the heart rhythm [28]. In our data, a higher CHA2DS2-VASc score in the study group could be explained by a significantly advanced age. Low LAA blood flow velocity, age and cardiovascular morbidity seem to be mutually affect each other. Therefore, their influence on patients with acute ischemic stroke or a cardiac disease should be investigated in further studies separately. Interestingly, a previous stroke was found more often in patients with LAA-HF. This seems to be hard to explain in the context of this study as there was no reliable information about the LAA blood flow velocity or the etiology of the previous strokes.

Our observations on cardiovascular morbidity can also be found in patients with left atrial (LA)-cardiopathy [16, 29, 30]. Owing to increasing evidence of overlapping risks for both stroke and AF in patients with LA-cardiopathy [30], low LAA blood flow velocity may be an early symptom of a progressive LA disease with AF as follows intermediate or end-stage stadium. There is also the possibility of subclinical paroxysmal AF and LAA-LF as a morphological manifestation of LA-dysfunction that persists even in sinus rhythm. Pacemaker studies have demonstrated that a stroke is not necessarily linked to a previous period of AF. In some cases, AF occurred even after the index stroke [31]. The high incidence of new-onset AF in the first year in our data could be explained by a closer patient monitoring after hospitalization and potentially hidden AF that has not been captured during hospital admission. Supposed cardioembolic stroke might be the result of LAA-dysfunction due to advanced LA-cardiopathy, or AF, or a (necessary) combination thereof that could be assessed with additional LAA-TEE data.

### Limitations

We searched for participants in a single-center design by identifying patients previously scheduled for a TEE examination. These patients had an indication for TEE, which could lead to a selection bias. Therefore, most of our patients had recently experienced an ischemic stroke or were in preparation for a TAVR. Both groups are at risk of AF [32, 33]. Although new-onset AF was not associated with TAVR or LAA-LF in our data, a cardiac intervention at baseline bears the risk of confounding our results on new-onset AF detection and new strokes. A small study revealed an increasing LAA blood flow velocity after TAVR [34]. Nonetheless, the restoration of LAA blood flow velocity could have an unrecognized influence on our observations. We also detected unknown AF at baseline by using an ECG-Holter device for 24–72 h. Studies have shown, that the detection rate with a single ECG-Holter duration of 24–72 h can be as low as 2.4–6.0% [8]. This could result in a certain number of patients in both groups having an undiagnosed or subclinical AF. Additionally, we recruited and followed many participants during the COVID-19 pandemic. A recent meta-analysis revealed a higher incidence of AF in patients who recovered from COVID-19, which could have influenced our findings [35]. Our results are based and interpreted with explorative data. Unfortunately, only half of our participants have reached the 36 months follow-up appointment.

## Conclusion

A low LAA blood flow velocity (*≤* 60 cm/s) in sinus rhythm is associated with an independently increased risk of new-onset AF, which resulted in higher rates of stroke at 3 years of follow-up. Furthermore, the risk of AF continuously increases as the LAA blood flow velocity decreases. Cardiovascular burden and advanced age were found to occur more frequently in patients with low LAA blood flow velocity, suggesting a closer monitoring of these patients. Therefore, additional simple LAA-TEE data could help to identify patients who could benefit from more accurate cardiac rhythm monitoring.

## Data Availability

The datasets used and/or analyzed during the current study are available from the corresponding author on reasonable request.

## Abbreviations

AF: Atrial fibrillation
BMI: Body mass index
CAD: Coronary artery disease
CKD: Chronic kidney disease
ECG: Electrocardiography
LA: Left atrium
LAA: Left atrial appendage
LAA-HF: Left atrial appendage– high flow ≥ 60 cm/s
LAA-LF: Left atrial appendage– low flow < 60 cm/s
LAVI: Left atrial volume index
LVEF: Left ventricular ejection fraction
NPV: Negative predictive value
NT-proBNP: Amino-terminal pro-brain-natriuretic peptide
PPV: Positive predictive value
ROC: Receiver operating characteristics
TAVR: Transfemoral aortic valve replacement
TEE: Transesophageal echocardiography
TTE: Transthoracic echocardiography
